# Barriers and facilitators to adopting health-promoting lifestyles in patients with psoriasis in China: a qualitative study using the COM-B and SEM

**DOI:** 10.3389/fpubh.2025.1695306

**Published:** 2025-11-26

**Authors:** Liyuan Xing, Huiqian Yu, Sisi Wang, Shuya Huang, Hongwei Liu, Jinghui Song

**Affiliations:** 1 Department of Nursing, Henan Provincial People's Hospital, Zhengzhou, China; 2 Department of Nursing, Zhengzhou University People's Hospital, Zhengzhou, China; 3 Henan Provincial Intelligent Nursing and Transformation Engineering Research Center, Zhengzhou, China; 4 Henan Provincial Key Medicine Laboratory of Nursing, Zhengzhou, China; 5 Dermatology Department, Henan Provincial People's Hospital, Zhengzhou, China; 6 Dermatology Department, Zhengzhou University People's Hospital, Zhengzhou, China

**Keywords:** psoriasis, barriers, facilitators, lifestyle, qualitative study

## Abstract

**Background:**

Psoriasis is an inflammatory skin disease that places a heavy burden on patients and society. Lifestyle can affect the occurrence and progression of psoriasis and its associated comorbidities.

**Objectives:**

To explore facilitators and barriers to health-promoting lifestyles among patients with psoriasis using the capability, opportunity, motivation behavior (COM-B) model and the Social Ecological Model.

**Methods:**

A descriptive qualitative study was conducted using semi-structured interviews with 16 patients recruited via a WeChat group of Psoriasis Patients' Home. Data were collected and analyzed using content analysis in NVivo software (version 14).

**Results:**

At the individual level, barriers included limited knowledge and awareness, excessive dietary restrictions, symptom burden, difficulty changing habits, ineffective behaviors, enjoying life in time, self-awareness of controllable conditions, food temptation, and inappropriate self-image; facilitators were disease knowledge, healthy diet attention, avoidance of side effects of medicine, effective behavioral change, proactive information seeking, self-feeling of aggravating skin lesions by bad habits, improved body image, self-pleasure, and overall health promotion. At the interpersonal level, barriers were social contact needs, while facilitators included peer and family support and patient role models. At the organizational/community level, barriers were work and life pressure and unhealthy workplace eating, whereas facilitators were community network support. At the societal level, barriers were stigmatization and lack of disease-specific health promotion guidance.

**Conclusions:**

This study highlights the multi-level factors influencing health-promoting lifestyles among patients with psoriasis and underscores the need for interventions that enhance individual capability and motivation while fostering supportive social and environmental contexts.

## Background

Psoriasis is a chronic inflammatory skin condition with a relapsing and remitting pattern, affecting 2% to 3% of the global population (1, 2). Although not life-threatening, it can cause substantial functional, psychological, and social impairment, significantly reducing health-related quality of life and placing a considerable burden on both patients and society (3).

In recent years, chronic disease management has shifted from a sole focus on symptom control toward the promotion of healthy lifestyles (4, 5). Unhealthy behaviors are known to increase both the risk and severity of chronic conditions, prompting growing interest in lifestyle modification as a means to improve health outcomes (6–8). A health-promoting lifestyle, comprising daily behaviors that enhance physical and mental well-being, has been shown to improve treatment adherence, quality of life, and prognosis in patients with chronic diseases (9–11).

Lifestyle factors play a particularly important role in psoriasis and its comorbidities. Stress, diet, smoking, alcohol consumption, and physical activity are not only associated with disease onset and progression but may also influence treatment response (12). Evidence from systematic reviews suggests that low-calorie diets and exercise programs can reduce psoriasis severity (7). Although no intervention trials have specifically tested alcohol abstinence or smoking cessation, observational studies have linked these behaviors to poorer treatment outcomes (13, 14). Despite these findings, many patients with psoriasis continue to smoke, drink alcohol, and lead sedentary lifestyles, exacerbating both the condition and its complications.

**Figure 1:**
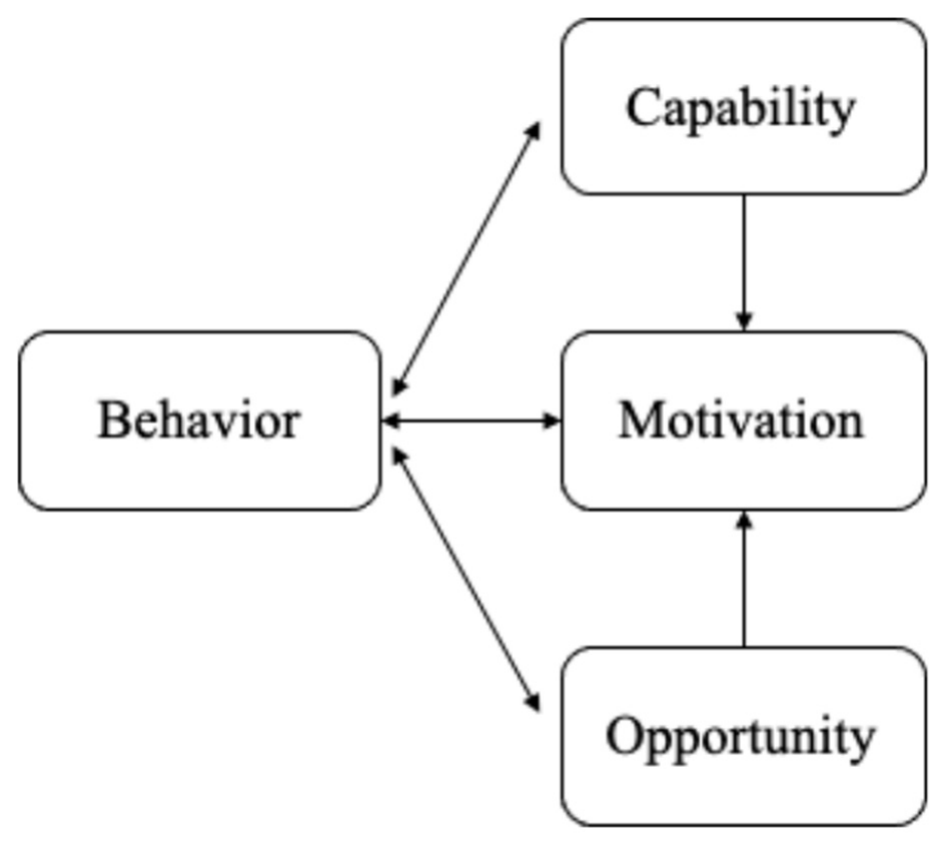
The COM-B model.

**Figure 2:**
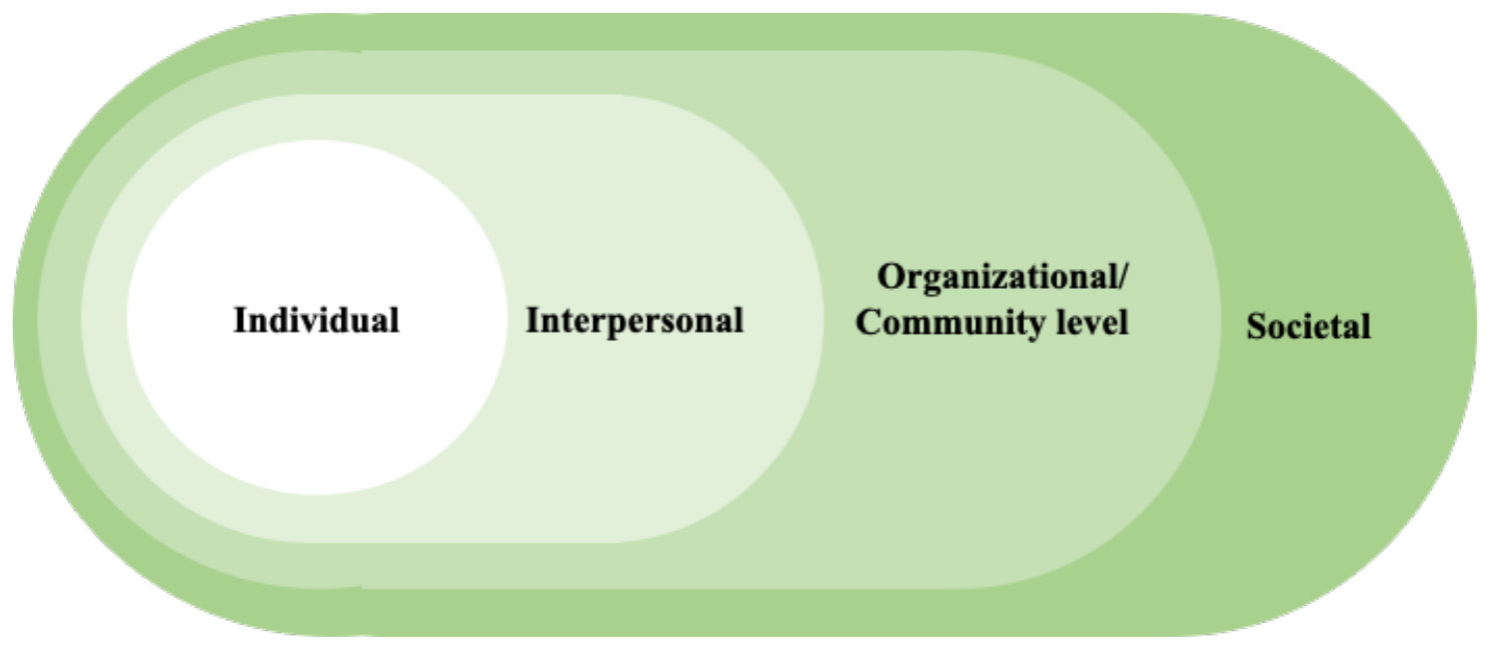
The social ecological model.

Adopting a health-promoting lifestyle is a complex behavioral process, especially for individuals with chronic conditions such as psoriasis. To better understand the mechanisms underlying such behaviors, theoretical frameworks are essential for identifying both barriers and facilitators. The capability, opportunity, motivation behavior (COM-B) model provides a systematic lens for examining health-related behaviors in healthcare contexts (15, 16). This model posits that engaging in a target behavior requires adequate capability (knowledge and skills), opportunity (social and environmental resources), and motivation (cognitive and emotional drivers) (Figure 1) (17). In parallel, the Social Ecological Model (SEM) highlights that individual behaviors are shaped by multiple levels of influence, including individual, interpersonal, organizational or community level, and societal factors (Figure 2) (18). By situating individual decision-making within broader social and structural contexts, SEM complements the mechanism-oriented focus of COM-B. Guided by the integration of these two frameworks, the present study explores the barriers and facilitators to adopting health-promoting lifestyles among patients with psoriasis in China.

## Methods

### Design

This was a descriptive qualitative study conducted in a tertiary hospital in central China from March to September 2023. This qualitative study adhered to the Standards for Reporting Qualitative Research (SRQR) to ensure methodological rigor.

### Participants and setting

This study included patients diagnosed with psoriasis for a minimum of 6 months who were at least 18 years of age and had no other chronic diseases. A purposive sampling strategy was adopted to achieve diversity in demographic characteristics. Participants were recruited from a WeChat-based psoriasis patient support group established in 2015 and managed by patient representatives in central China. After receiving ethical approval and permission from the group managers, the researcher posted a recruitment notice in the group. Interested patients were provided with detailed information about the study, and those who agreed to participate signed an informed consent form. Data collection continued until meaning saturation was reached—that is, when no new concepts relevant to the research objectives emerged from subsequent interviews (19).

### Data collection

Online, one-on-one interviews were conducted at mutually agreed-upon times by a researcher experienced in qualitative methods. Before the interview, participants provided demographic information, including gender, age, marital status, place of residence, education level, and psoriasis type and duration. The semi-structured interview guide was developed based on the COM-B model and the Social Ecological Model, supplemented by findings from previous studies on lifestyle behaviors in chronic diseases (20, 21). The guide was reviewed by two experts in behavioral nursing and dermatology for content validity. A pilot interview was conducted with one psoriasis patient to ensure question clarity and relevance; minor wording adjustments were made before formal data collection. All interviews were audio recorded and transcribed verbatim. The patients were informed that they could request to switch off the recorder and withdraw at any time.

### Data analysis

Data were collected and analyzed simultaneously using content analysis. The transcripts were uploaded to NVivo software (version 14) and read several times. The analysis was guided by the COM-B model and the SEM. Specifically, the COM-B model was used to categorize determinants of behavior into three domains: capability, opportunity, and motivation, while SEM provided a multilevel perspective, considering individual, interpersonal, organizational or community level, and societal influences. The researcher learned once again and discussed the COM-B model and SEM to understand it before in-depth coding. The transcripts were inductively coded by the two researchers separately. Any doubt about some coding was resolved through discussions or consultations with other researchers to reach an agreement. After reading the transcripts repeatedly, the author coded them. All codes were then mapped to the relevant COM-B components and SEM levels to provide a comprehensive, multilevel understanding of factors influencing health-promoting behaviors. For example, “limited knowledge and awareness” was coded as psychological capability at the individual level, while “peer and family support” was mapped to social opportunity at the interpersonal level. Similarly, “work and life pressure” was categorized as physical and social opportunity barriers at the organizational/community level, and “stigmatization” represented a societal-level social opportunity constraint. This dual-mapping approach enabled systematic interpretation of behavioral determinants across ecological layers.

### Rigor and trustworthiness

To ensure the trustworthiness of this qualitative study, we adopted multiple techniques. Two researchers independently coded the transcripts and discussed coding results to reach consensus and a third researcher was consulted when discrepancies arose. Member checking was conducted by sharing coded excerpts with participants to verify accuracy. Throughout the process, reflexive practices were used to bracket assumptions and ensure interpretations reflected participants' perspectives. Transferability was enhanced by providing detailed descriptions of the research context, participant characteristics, and representative quotations.

## Results

### Study participant characteristics

A total of 16 patients (10 men and 6 women) with different types of psoriasis aged 23–69 years participated in this study. The participants included different ranges of marital status, education levels and residence. The disease duration ranged from 3 to 35 years (see Table 1). 14 barriers and 13 facilitators were revealed in relevant domains and mapped onto the COM-B components. Furthermore, the SEM was applied to stratify these factors into individual, interpersonal, organizational or community, and societal levels, highlighting the multilevel influences on health-promoting lifestyle behaviors (see Table 2).

**Table 1:** Demographic profiles of participants.

**Characteristic**	***N***
**Gender**	
Male	10
Female	6
**Age (years)**	
20–29	3
30–39	6
40–49	1
50–59	4
60–69	2
**Marital status**	
Single	2
Married	14
**Residence**	
Urban	12
Rural	4
**Education level**	
Junior high school and below	6
High school	2
College	5
Graduate and above	3
**Disease type**	
Plaque psoriasis	12
Erythrodermic psoriasis	2
Psoriatic arthritis	2
**Disease duration**	
1–5	1
6–10	2
11–15	4
16–20	5
>20	4

**Table 2:** Multilevel barriers and facilitators to health-promoting lifestyles.

**SEM level**	**COM-B domain**	**Barriers**	**Facilitators**
Individual	Capability	- Lack of knowledge and awareness of a health-promoting lifestyle; - Excessive dietary restrictions; - Symptom burden.	- Knowledge of the disease; - Attention to a healthy and scientific diet.
	Motivation	- Difficulty in changing habits; - Non-effective behavior change; - Enjoying life in time; - Self-awareness of controllable conditions; - Temptation of food; - Inappropriate self-image.	- Avoidance of side effects of medicine; - Effective behavioral change; - Proactive access to health-promoting information resources; - Self-feeling of aggravating skin lesions by bad habits; - Improving body image; - Self-pleasure; - Overall health promotion.
Interpersonal	Opportunity	- Social contact needs	- Peer support; - Family support, especially healthy diet attention; - Real cases of patients
Organizational/Community	Opportunity	- Work and life pressure; - Eating at the workplace.	- Community network support.
Societal	Opportunity	- Stigmatization experience; - Limited availability of disease-specific health promotion guidance.	

### Individual level

#### Capability

##### Barrier—lack of knowledge and awareness of a health-promoting lifestyle

Most participants reported insufficient knowledge and awareness of health-promoting lifestyles, which limited their ability to make informed decisions. They did not recognize the importance of a health-promoting lifestyle for disease control. A participant noted:

“*I think good living habits are to go to bed early and get up early, and live regularly and feel comfortable. That is enough.”* (N5)

##### Barrier—excessive dietary restrictions

Participants demonstrated limited knowledge about how to manage their diet appropriately. Many reported being told that numerous foods should be avoided to prevent disease progression, which led to excessive dietary restrictions. In particular, most participants believed that consuming beef or lamb would worsen their condition, a perception influenced by traditional Chinese medicine practitioners or advice from others. One participant stated:

“*After the first diagnosis of psoriasis, people I knew said that you cannot eat fish, you cannot eat shrimp, you cannot eat kelp and chicken, so I have been avoiding certain foods for more than ten years*.” (N11)

##### Barrier—symptom burden

In severe cases, psoriasis lesions led to chapped skin, pain, and even bleeding, which significantly restricted participants' daily activities and physical movement. These symptoms not only reduced their ability to engage in exercise or outdoor activities but also undermined their confidence in maintaining health-promoting behaviors. One participant expressed the following:

“*My onset site is in the calf. When my condition worsens, I feel dry and painful in the skin lesion. In that situation, I feel uncomfortable during daily activities.”* (N12)

##### Facilitator-knowledge of the disease

Participants who had knowledge of disease management and recognized the importance of health-promoting lifestyles were more likely to adopt healthy behaviors to control their condition. They emphasized that lifestyle management was as crucial as medical treatment. As one participant noted:

“*I know that scientific treatments are important for disease control. A regular daily routine can help me manage my condition.”* (N1)

##### Facilitator-attention to a healthy and scientific diet

Several participants highlighted the value of dietary management based on personal observation, rather than rigidly adhering to generalized restrictions. They reported paying close attention to their skin's reaction to specific foods and making individualized dietary adjustments. One participant explained:

“*It's often said that this disease requires a restricted diet, but I don't blindly follow others' advice. I pay attention to how my skin reacts to different foods. If I notice a sensitivity to a certain food, I avoid it. After some time, I might try it again, and if my skin reacts again, I know to avoid it in the future.”* (N13)

#### Motivation

##### Barrier—difficulty in changing habits

Participants demonstrated difficulties in changing entrenched habits, particularly those related to smoking and drinking. They found it challenging to manage long-standing routines.

“*I have been smoking for many years, and I am addicted to smoking, so it is difficult to quit. It is too difficult for me to manage my lifestyle.”* (N5)

##### Barrier—non-effective behavior change

Some participants acknowledged the benefits of adopting a healthy lifestyle but expressed disappointment when lifestyle modifications did not lead to significant improvements in their condition. This reduced their motivation to persist.

“*I used to quit drinking because I thought I could control the disease better without wine, but I found that it was not particularly useful, so I started drinking occasionally again.”* (N5)

##### Barrier—enjoying life in time

Several participants perceived that focusing too much on lifestyle restrictions reduced their quality of life. They prioritized immediate enjoyment, even if it conflicted with health-promoting behaviors. One explained:

“*I do not pay special attention to the way I live my daily life now. Life is short. If I do not smoke and drink, I feel like I am missing something in my life.”* (N13)

##### Barrier—self-awareness of controllable conditions

Participants showed low perceived necessity for behavioral change. When their disease was stable and not actively progressing, they believed there was no need to improve their lifestyle, which weakened their motivation to adopt or sustain health-promoting practices. An individual described:

“*My disease is controlled well. There are not many skin lesions on my body now. I have not considered changing my existing lifestyle.”* (N13)

##### Barrier—temptation of food

Participants admitted difficulty resisting attractive but unhealthy foods, particularly fried or high-fat options. One participant described:

*“I like to eat greasy things which taste more fragrant, and I cannot resist temptation.”* (N11)

##### Barrier –inappropriate self-image

Participants expressed dissatisfaction with their physical appearance, leading to a negative self-image and avoidance of social activities. One participant shared:

“*I wear long sleeves and socks all year round. I never wear collarless clothes. Skin lesions greatly affect my appearance, and I do not want to expose them.”* (N3)

##### Facilitator—avoidance of side effects of medicine

Awareness of the potential side effects of long-term medication use motivated some participants to adopt healthier lifestyles as an alternative or complementary strategy. One participant explained:

“*I've been taking Chinese or Western medicine for a long time. For me, taking medicine is more painful than exercising. Chinese medicine is a bit better, but with Western medicine, I have to regularly check my liver and kidney functions every two or three months because these drugs have side effects. So I think it's better to exercise. I really don't want to take medicine.”* (N6)

##### Facilitator—effective behavioral change

Participants who experienced positive outcomes from lifestyle modifications were more likely to sustain these behaviors. For example, one participant mentioned:

“*I feel better when I live a regular life. If you live irregularly, for example, staying up late or in a bad mood, depressed and anxious, this will aggravate the condition. There will be many skin lesions on the body quickly.”* (N5)

##### Facilitator—proactive access to health-promoting information resources

Several participants actively sought lifestyle-related health information, particularly through online platforms, which enhanced their motivation to engage in healthier behaviors. One participant shared:

“*I always search in self-media and online to obtain more knowledge to understand how lifestyle impacts the disease.”* (N4)

##### Facilitator—self-feeling of aggravating skin lesions by bad habits

Participants recognized that unhealthy habits, such as smoking or drinking, directly worsened their skin condition, which motivated them to reduce these behaviors. One individual described the following:

“*Smoking and drinking alcohol can aggravate my condition. Smoking is particularly noticeable. If I smoke now, I may quickly rebound or worsen my condition in two to three days.”* (N13)

##### Facilitator—improving body image

Lifestyle changes such as regular exercise and a healthy diet were also associated with improved appearance and body image. For example, one individual mentioned:

“*I want to lose weight to control my weight, and I will look better in clothes.”* (N14)

##### Facilitator—self-pleasure

Engaging in exercise provided participants with enjoyment and improved mood, which served as a strong motivator for maintaining healthy behaviors. One individual described the following:

“*Some of my current lifestyle is for my own good mood. For example, running is pleasant, that is, there is a happy mood after sweating.”* (N5)

##### Facilitator—overall health promotion

It was recognized that maintaining a healthy lifestyle was not only about disease management but also about improving overall health, with one participant expressing the following:

“*I am not sure whether exercise is good for this disease. Sometimes I can feel the benefits and sometimes not. I think exercise is helpful to my overall body.”* (N6)

### Interpersonal level

#### Opportunity

##### Barrier—social contact needs

Participants described the difficulty of refusing cigarettes or alcohol in social settings, particularly during meals or business gatherings, where drinking was viewed as a cultural norm. For example, one explained:

“*It is inevitable that there will be socialization in life, and sometimes I go out to eat with clients, I have to drink wine.”* (N16)

##### Facilitator—peer support

Having peers with similar conditions was seen as beneficial for sustaining lifestyle changes, as they could encourage and support one another. One participant noted:

“*If there is a group of patients in a place, which can hold some activities on a daily basis, people with the same disease can communicate with each other and encourage each other to improve their behavior. It is a bit difficult to improve health promotion lifestyle by oneself.”* (N8)

##### Facilitator—family support, especially healthy diet attention

Family members play an important role in promoting healthy eating habits by encouraging and preparing nutritious meals. Their attention to dietary choices can help patients maintain a balanced lifestyle. One participant noted:

“*If I go home to eat, I can basically eat healthily because my wife has healthier eating habits. She always cooks food with low oil and low fat.”* (N13)

##### Facilitator—real cases of patients

Participants reported that hearing authentic experiences from other patients, especially examples of how lifestyle improvements contributed to disease control, motivated them to adopt and maintain health-promoting behaviors. One participant shared:

“*I met a female patient in the patient group. She told me that she insisted on exercising every day before, and the skin lesions gradually disappeared. After the disease improved, it basically did not affect her normal life. Then I started to do exercise.”* (N6)

### Organizational/Community level

#### Opportunity

##### Barrier—work and life pressure

Most participants reported being overwhelmed by daily work demands and feeling physically exhausted. Some mentioned irregular rest during night shifts. Limited time and fatigue prevented them from engaging in exercise or other health-promoting behaviors. One participant expressed:

“*Subject to life and work, it is difficult to keep a regular schedule, and I do not want to exercise when I am busy with work.”* (N5)

##### Barrier—eating at the workplace

Some participants explained that they usually ate meals provided at the workplace or ordered takeout while on duty. As a result, healthy eating was often compromised. One participant stated:

“*When I go to work, I eat work meals. I eat whatever the workplace restaurant cooks. I cannot just ask the chef to do so, and I have not thought about asking the chef to pay attention to the combination of ingredients, low salt and low oil.”* (N12)

##### Facilitator—community network support

Psoriasis is often misunderstood by the public as a contagious disease, leading to stigma and discrimination. Participants emphasized the importance of understanding and acceptance from their community and close contacts, expressing a desire to be treated without prejudice. One participant shared:

“*I think it is important for others to understand psoriasis. The public should not discriminate against patients and not point fingers at us. My family, friends, and people near me know this disease well. They would not judge me, which gave me energy.”* (N8)

### Societal level

#### Opportunity

##### Barrier—stigmatization experience

Most participants experienced stigmatization because of skin lesions, which made them reluctant to engage in health-promoting behaviors, such as exercise in public. For example, one participant mentioned:

“*I do not want to exercise in public. There are always some strange looks from the public when they see the skin lesions on my body.”* (N2)

##### Barrier—limited availability of disease-specific health promotion guidance

Most participants searched online for disease information. Not much content is available online about the health-promoting lifestyles of patients with psoriasis. Doctors occasionally tell patients about behaviors that they should improve in their daily lives. However, this information is limited.

“*I often search online to learn about this disease. I feel that there is no other way to learn more about psoriasis. The hospital has too little publicity about disease education. I got less information from the hospital.”* (N8)

## Discussion

This study explored barriers and facilitators to adopting health-promoting lifestyles among Chinese patients with psoriasis, based on the COM-B model and SEM. The findings indicate that influences across multiple levels shape behavior change, but the distribution is uneven: facilitators and barriers were identified at the individual, interpersonal, and organizational levels, whereas only barriers emerged at the societal level.

In the COM-B model, capability and motivation were primarily reflected at the individual level. Many patients lacked knowledge about health-promoting lifestyles or believed that lifestyle change was unnecessary when symptoms were controllable, which is similar to previous studies (22, 23). People who do not possess the necessary knowledge about health behaviors may lack the cognitive skills required to understand the importance or potential consequences of behaviors (20, 24, 25). Adebajo and Akintayo reported that poor knowledge of the disease can decrease treatment adherence (26). Lack of knowledge also affected dietary practices, as many patients reported restricted diets inconsistent with evidence-based guidelines. This highlights the need for clinicians to provide updated guidance when delivering nursing care.

Physical capability was another barrier. Psoriasis symptoms such as itching, burning, pain, and, in severe cases, limited joint mobility due to psoriatic arthritis, impede exercise and other physical activities (24). This is consistent with previous findings in cancer survivors, where physical symptoms limited engagement in healthy behaviors. Effective symptom management by healthcare providers is therefore crucial to facilitate lifestyle change.

Motivational factors were also critical. Difficulty resisting food temptation, breaking entrenched habits, and coping with symptom burden undermined motivation. Body image concerns served as both barrier and facilitator. While negative self-perception reduced willingness to engage in activities exposing skin lesions, recognition that exercise could improve appearance and health motivated some patients (13). Similarly, instant gratification could hinder adherence to health-promoting behaviors, yet self-pleasure from positive health practices, such as exercising or preparing nutritious meals, acted as a facilitator (27). Individual differences in perceptions and trial-and-error processes indicate that personalized interventions and shared decision-making are essential. At the same time, facilitators were evident. Some patients acknowledged that lifestyle change could improve overall health, prevent medication side effects, and enhance body image and self-pleasure. These findings suggest that strengthening patients' knowledge and fostering reflective motivation may be key to sustaining lifestyle change.

Opportunities were largely embedded in interpersonal and organizational contexts, consistent with SEM. Social support facilitated behavior change, peer encouragement, family involvement, and examples of successful lifestyle modification promoted adherence (20, 21, 24). Conversely, social stigma, communal eating practices, and social expectations sometimes conflicted with self-management. Stigmatization reduced self-esteem, leading to shame or self-blame, which discouraged engagement in healthy behaviors (3, 27). Addressing these psychosocial barriers may require stress management programs and community outreach to reduce stigma and foster supportive social networks. At the organizational and societal level, barriers included limited access to reliable health information, workplace eating constraints, and life–work pressures. Patients often resorted to online searches that exposed them to incorrect information (26), consistent with previous study (8). It emphasizes the importance of providing structured, evidence-based resources and creating supportive environments within healthcare and workplace settings.

The findings indicated an absence of facilitators at the societal level. Patients primarily reported stigmatization, which increased psychological burden and discouraged engagement in healthy lifestyles (28). This pattern likely reflects broader limitations in public health systems and socio-cultural contexts, where policies, media representation, and public awareness have not provided adequate support for psoriasis management. Within the COM-B model and the SEM, this highlights insufficient opportunity at the macro level. Evidence from other chronic disease populations suggests that societal-level facilitators can play a meaningful role in promoting healthy behaviors. For instance, in cardiovascular disease and diabetes management, health policies supporting structured rehabilitation programs, public education campaigns, and accessible societal support resources have been reported to encourage lifestyle modification (23, 29). These findings imply that macro-level strategies, such as anti-stigma initiatives, enhanced public education, and supportive health policies, may facilitate lifestyle modification among individuals with psoriasis. Future studies should explore societal and policy-level mechanisms that could support lifestyle change in this patient group.

By combining COM-B model and the SEM, this study highlighted both the intra-individual mechanisms of behavior (capability and motivation) and external opportunities situated at multiple levels. The imbalance observed, where capability and motivation clustered at the individual level while opportunities spanned interpersonal, organizational, and societal domains, underscores the need for multi-level interventions. Practical implications include strengthening patient education and correcting misconceptions at the individual level, fostering supportive family and workplace environments, and advancing societal strategies such as health promotion campaigns and stigma reduction.

The strengths of this study lie in its theoretical foundation using the intergration of the COM-B model and the SEM, which provides a comprehensive lens for understanding behavior change in chronic disease management. This study represents novel qualitative insights into barriers and facilitators to adopting healthy lifestyles among Chinese patients with psoriasis, filling an important gap in both dermatology and behavioral health literature. The use of in-depth interviews allowed for a rich and nuanced understanding of patients' perceptions and experiences. However, several limitations should be acknowledged. The majority of participants in this study had plaque psoriasis, which reflects the epidemiological distribution of psoriasis, as plaque psoriasis is the most common clinical subtype. Nevertheless, the findings may have limited transferability to patients with other subtypes. Future research should include more diverse clinical phenotypes to validate and extend these results. Participants were recruited through a WeChat patient community, possibly excluding individuals with limited digital access or lower health literacy, introducing potential selection bias. In addition, data were derived solely from patient interviews without triangulation from other sources or perspectives such as healthcare professionals. Further mixed-methods or quantitative studies involving diverse populations and multiple stakeholders are warranted to validate and extend these findings.

## Conclusion

This qualitative study, guided by the COM-B model and the Social Ecological Model, identified multilevel barriers and facilitators to adopting health-promoting lifestyles among Chinese patients with psoriasis. These findings emphasize the need for multi-level interventions, including enhancing patient education and motivation, fostering supportive interpersonal and organizational contexts, and addressing stigma and promoting public awareness at the societal level. Collectively, these strategies hold promise for enabling patients with psoriasis to maintain health-promoting lifestyles and achieve better quality of life.

## Data Availability

The datasets used and analyzed during the current study are available from the corresponding author on reasonable request.
